# Co-culturing of follicles with interstitial cells in collagen gel reproduce follicular development accompanied with theca cell layer formation

**DOI:** 10.1186/1477-7827-9-159

**Published:** 2011-12-17

**Authors:** Saori Itami, Keiko Yasuda, Yuka Yoshida, Chiyuki Matsui, Sachie Hashiura, Atsushi Sakai, Satoshi Tamotsu

**Affiliations:** 1 School of Natural Science and Ecological Awareness, Graduate School of Humanities and Sciences, Nara Women's University, Kitauoyahigashi-machi Nara 630-8506, Japan; 2 Department of Biological Sciences, Faculty of Science, Nara Women's University, Kitauoyahigashi-machi, Nara 630-8506, Japan

## Abstract

**Background:**

The mechanism of theca cell layer formation in mammalian ovaries has not been elucidated; one reason is that there is no follicle culture system that can reproduce theca cell layer formation in vitro. Therefore, a three-dimensional follicle culture system that can reproduce theca cell layer formation is required.

**Methods:**

A collagen gel was used in the follicle culture system. To determine the optimum conditions for follicle culture that can reproduce theca cell layer formation, the effects of hormonal treatment and cell types co-cultured with follicles were examined. In addition, immunohistochemistry was used to examine the properties of the cell layers formed in the outermost part of follicles.

**Results:**

Follicles maintained a three-dimensional shape and grew in collagen gel. By adding follicle-stimulating hormone (FSH) and co-culturing with interstitial cells, the follicles grew well, and cell layers were formed in the outermost part of follicles. Immunohistochemistry confirmed that the cells forming the outermost layers of the follicles were theca cells.

**Conclusion:**

In this study, follicle culture system that can reproduce theca cell layer formation *in vitro *was established. In our opinion, this system is suitable for the analysis of theca cell layer formation and contributes to our understanding of the mechanisms of folliculogenesis.

## Background

The follicles in mammalian ovaries are composed of a single oocyte, granulosa cells, and theca cells. Although the theca cell layer is not recognizable in primordial follicles, it is identifiable from the stage of secondary follicles onward. It is considered that theca cells play roles in the physical maintenance of follicle structure by constructing cell layers around the basement membrane. In addition, theca cells are crucial for folliculogenesis because theca cells and granulosa cells cooperatively synthesize steroid hormones that promote folliculogenesis [1-3]. However, knowledge of theca cells, particularly theca cell layer formation, is less than that of granulosa cells and oocytes. For example, the origin of theca cells has not been fully elucidated. Although it is believed that ovarian interstitial cells gather around follicles and differentiate into theca cells [1,2], how and when these cells forming the theca cell layer appear around follicles and result in layered organization has not been clarified. Because it is difficult to follow the behavior of interstitial and theca cells *in vivo*, a follicle culture system that can reproduce theca cell layer formation *in vitro *is necessary.

To determine the function of ovarian cells, several cell culture systems, including those co-culturing granulosa and theca cells, have been developed [4-9]. In addition, follicle cultures have been examined to elucidate the mechanisms of folliculogenesis [10-13]. However, in liquid media, follicles are attached to the bottom of culture dishes and cannot maintain the three-dimensional shape observed *in vivo*. Some culture systems have been developed to resolve this problem. For example, when granulosa cells and endothelial cells were co-cultured in a multi well plate with a nonadherent round-bottom, these cells did not spread on the bottom and formed spheroids [14]. In another system, Lenie et al. [15] cultured follicles in a medium containing a low concentration of serum, followed by transfer to a new dish in order to avoid cell attachment to the culture dish. In this culture system, granulosa cells remained around the oocyte, and the follicles maintained their structure. Another method to retain the three-dimensional structure of the follicles is to embed them in a gel during culture. In mice, the addition of type I collagen in alginate-based matrices promoted follicular growth better than the addition of other extracellular matrices [16]. In addition, it was reported that bovine [17] and mouse [18] follicles maintained their structure during culture when embedded in a collagen gel. However, the follicles remained attach the ovarian tissue or were already formed multiple theca cell layers from the start of culture. In this culture system, theca cell layer was observed, but that were already formed. These culture systems were therefore insufficient for the examination of theca cell layer formation. In this study, we established a three-dimensional follicle culture system that was suitable for the examination of theca cell layer formation.

## Methods

### Animals

Female ICR mice (Japan SLC, Inc., Shizuoka, Japan) were maintained under controlled light conditions (14 h light, 10 h dark) and were given food and water ad libitum. The day of birth was designated as day 0. On day 1, each mother was left with eight pups in order to equalize the growth of pups between litters. Our investigations were conducted in accordance with the Animal Care Committee of Nara Women's University.

### Chemicals and antibodies

The cell culture chemicals used in this study included Dulbecco's modified Eagle's medium (DMEM; Nissui Pharmaceutical Co., Ltd., Tokyo, Japan), fetal bovine serum (FBS; Gibco BRL/Invitrogen Corp., Carlsbad, CA, USA), penicillin and streptomycin (Nacalai Tesque Inc., Kyoto, Japan), collagenase (Wako Pure Chemical Industries, Ltd., Osaka, Japan), DNase (Roche Diagnostics Corp., Indianapolis, IN, USA), type I collagen (Cell matrix Type I-A; Nitta Gelatin Inc., Osaka, Japan), and human follicle-stimulating hormone (hFSH; Acris Antibodies GmbH, Herford, Germany).

The primary antibodies used in this study included the rabbit anti-mouse laminin antibody (Harbor Bio-Products, Norwood, MA, USA), rabbit anti-mouse fibronectin antibody (Biogenesis Nutraceuticals. Inc, Mill Creek, WA, USA), goat anti-mouse Flk-1 antibody (R&D Systems, Inc, Minneapolis, MN, USA), rat anti-mouse F4/80 antibody (Serotec Morphosys UK Ltd, Oxford UK), goat anti-mouse CYP17A-1 antibody (Santa Cruz Biotechnology, Inc., Santa Cruz, CA, USA), rat anti-mouse THY1 antibody (Biomeda, Foster City, CA, USA), rabbit anti-mouse collagen type IV antibody (Cosmo Bio Co., Ltd., Tokyo, Japan), and rat anti-mouse tenascin antibody (Sigma-Aldrich Corporation, St. Louis, MO, USA). The secondary antibodies used were donkey anti-rabbit IgG labeled with Alexa Fluor 594, donkey anti-goat IgG labeled with Alexa Fluor 488, goat anti-rat IgG labeled with Alexa Fluor 488, rabbit anti-goat IgG labeled with Alexa Fluor 594, (Molecular Probes/Invitrogen Corp., Carlsbad, CA, USA), and goat anti-rabbit biotinylated IgG (Vector Laboratories, Inc., Burlingame, CA. USA).

### Three-dimensional follicle culture system

#### Isolation of interstitial cells

Interstitial cells were isolated from the ovaries of 3-week-old mice. The mice used in one experiment were about 20 mice. The ovaries were isolated from connective tissues under a stereomicroscope and then collected in culture medium (DMEM with 10% FBS, 100 U/ml penicillin, 0.1 mg/ml streptomycin, and 100 ng/ml hFSH). The follicles on the surface of the ovaries were punctured with fine tweezers to remove granulosa cells and oocytes. These punctured ovaries were cut into 1-mm^3 ^fragments using scissors in a culture medium containing 0.2% collagenase and 0.1% DNase and were then pipetted to facilitate cell dispersion. The suspension of ovarian fragments was incubated at 37°C for 20 min and pipetted at the 10th and 20th min. Ovarian fragments, follicles, and oocytes that could not be removed by puncturing with fine tweezers were filtered through a series of nylon meshes (pore size order, 155; 82; 40; 20; and 10 μm). The resulting cell suspension was centrifuged at 250 × g for 5 min, and the supernatant removed. To reduce the effects of collagenase to a negligible level, the aforementioned washing procedure was repeated four times. Finally, the cells were suspended in culture medium, and the number of viable cells counted by trypan blue-exclusion test.

#### Isolation of granulosa cells

Granulosa cells were isolated from the ovaries of 3-week-old mice. The mice used in one experiment were about 20 mice. The follicles on the surface of the ovaries were punctured with fine tweezers and the released cells collected. The cell suspension was filtered through a series of nylon meshes (pore size order, 82; 40; 20; and 10 μm), centrifuged at 250 × g for 5 min, and the supernatant then removed. Finally, the cells were suspended in culture medium and the number of viable cells counted.

#### Isolation of preantral follicles

Preantral follicles (diameter, approximately 140 μm) were obtained from 10- to 14-day-old female mice. The ovaries were collected in the culture medium, and the follicles were mechanically isolated from the ovaries under a stereomicroscope using a 27-gauge needle fitted to a 1-ml syringe barrel. The follicles were selected using a mouth-operated glass fine pipette and were then transferred to a culture dish.

#### Follicle culture system using collagen gel

A collagen gel was prepared according to the manufacturer's instructions. Type I collagen and DMEM were mixed, and the pH adjusted to 7.4 with reconstitution buffer. The collagen gel was supplemented with 10% FBS, 100 U/ml penicillin, 0.1 mg/ml streptomycin, and 100 ng/ml hFSH. The collagen gel solution (50 μl) was poured into each well of a 96-well culture plate (Microplate 96 Well with Lid; AGC TECHNO GLASS Co., Ltd., Chiba, Japan) and was allowed to solidify at 37°C in 5% CO_2 _in air and 100% humidity. Interstitial cells were inoculated onto the gel (6 × 10^4 ^cells/well) and incubated for 1 h. Follicles were then placed on the interstitial cell layer and incubated overnight. The medium was removed, followed by the addition of collagen gel solution (50 μl) to encapsulate the follicles. After the collagen solution solidified, 50 μl of culture medium was then added. In several experiments, the follicles were cultured in the absence of interstitial cells or in the presence of granulosa cells instead of interstitial cells. FSH was added in all follicle culture unless the case that describe without FSH. The follicles were cultured for 5 days at 37°C in 5% CO_2 _in air and 100% humidity. During the culture period, the culture medium was changed every 2 days, and photographs of the follicles taken every day to check for follicle survival and measure their diameters. Follicle diameters (at basement membrane) were measured times per follicle to calculate the average.

To evaluate the morphology of follicles in the collagen gel, vertical and horizontal diameters were measured using histological methods. The follicles were fixed with 4% paraformaldehyde in PBS (PFA) (pH 7.4) or Bouin's solution at the end of the culture, embedded horizontally, and sectioned serially (thickness, 7 μm). The horizontal diameters of the follicles corresponded to the maximum diameters in the serial sections, while the vertical diameters were estimated by the number of serial sections.

### Cell culture

Interstitial and granulosa cells (2 × 10^4 ^cells) were inoculated on a 12-mm-diameter area of a 35-mm glass base dish (AGC TECHNO GLASS Co., Ltd., Chiba, Japan) and incubated for 2-3 h. The cells were either fixed with 4% PFA or left unfixed and then dried. These samples were used for the immunohistochemical detection of theca cell markers in order to investigate the purity of isolated interstitial cells.

### Immunohistochemistry

Immunohistochemistry was performed as described previously [19].

#### Preparation of sample

Briefly, isolated mouse ovaries or cultured follicles embedded in collagen gel were fixed with 4% PFA or Bouin's solution or left unfixed. The fixed or unfixed samples were embedded in embedding medium for frozen tissue specimens (O.C.T. compound; Sakura Finetek Japan Co.,Ltd., Chiba, Japan), frozen in liquid nitrogen, and sectioned (thickness, 7 μm) using a cryostat (Bright Instrument Co. Ltd., Cambridgeshire, UK) at -25°C. For paraffin sections, the fixed samples were dehydrated in a series of alcohol (50, 70, 80, 90, 95, and 100%), immersed in xylol, embedded in paraffin, and then sectioned (thickness, 7 μm). Suitable fixation and sectioning conditions were selected for each of the respective antigen properties.

#### Immunofluorescence

These procedures were performed at room temperature. In order to reduce nonspecific binding, the sections were incubated for 1 h with 10% normal serum of the animals of each secondary antibody in PBS. The sections were washed with PBS and incubated overnight with each primary antibody. After washing with PBS, the sections were incubated for 3 h with each appropriate secondary antibody. Finally, the cell nuclei were stained with DAPI (Nacalai Tesque, Inc., Kyoto, Japan). The combination of the primary and secondary antibodies used in this study is shown in Table 1.

**Table 1:** Combination of the first and secondary antibodies and fixation conditions used in immunohistochemistry

Antigen	First antibody	Secondary antibody	Fixation
F4/80	rat anti-mouse F4/80	goat anti-rat IgG Alexa Fluor 488	4% PFA
Flk-1	goat anti-mouse Flk-1	donkey anti-goat IgG Alexa Fluor 488	4% PFA
fibronectin	rabbit anti-mouse fibronectin	goat anti-rabbit IgG biotinylated	non-fixed
laminin	rabbit anti-mouse laminin	donkey anti-rabbit IgG Alexa Fluor 594	4% PFA
tenascin	rat anti-mouse tenascin	goat anti-rat IgG Alexa Fluor 488	4% PFA
collagen IV	rabbit anti-mouse collagen IV	donkey anti-rabbit IgG Alexa Fluor 594	4% PFA
THY1	rat anti-mouse THY1	goat anti-rat IgG Alexa Fluor 488	4% PFA
CYP17A-1	goat anti-mouse CYP17A-1	rabbi anti-goat IgG Alexa Fluor 594	Bouin's solution

#### ABC staining

These procedures were performed at room temperature. The sections were washed with PBS and incubated with 0.3% H_2_O_2_/methanol to remove endogenous peroxidase activity. To reduce nonspecific binding, the sections were incubated for 1 h with 10% normal serum of the animals of each secondary antibody in PBS. The sections were then incubated overnight with the primary antibody. After washing with PBS, the sections were incubated for 3 h with the secondary antibody, followed by 1 h incubation with the avidin-biotin peroxidase complex and visualization of the signals by 3, 3'-diaminobenzidine tetrahydrochloride. Finally, the cell nuclei were stained with methyl green.

Negative controls were treated with 0.5% BSA/PBS, instead of the primary antibodies, to assess nonspecific staining, and it was confirmed that the specific signals were not observed. The samples were examined using an Olympus BX51 fluorescent light microscope (Olympus Corp., Tokyo, Japan). For precise observation, a confocal laser scanning microscope system was also used (Nikon ECLIPSE Ti; Nikon Corporation, Tokyo, Japan). As we observed autofluorescence in the ovaries, a WIB long-pass filter cube was used for observing fluorescent samples. The use of this cube allowed us to distinguish significant signals (green signals) from autofluorescent ones (yellow signals from red blood cells).

### HE (haematoxylin-eosin) staining

To examine the morphology of the follicles, the sections were stained with Mayer's haematoxylin and eosin solution.

### Statistical analyses

In order to clarify the effect of FSH on the development of follicles and oocytes, the diameters of the follicles and the oocytes were measured using a micrometer. These were compared between the treatment group and the untreated control group. In addition, the effect of co-culture with interstitial cells on follicle growth was also examined. When the normality of residuals and homoscedasticity were confirmed, the t-test was performed. When the residuals were not normally distributed even after various (such as log or square-root) transformations were performed, the Mann-Whitney U test was used for comparisons between two groups. We focused the statistical tests on the relative sizes of follicles and oocytes on day 3 and day 5 to minimize the number of comparisons. Furthermore, to deal with type I (false positive) error due to multiple comparisons, Bonferroni correction was conducted by lowering the significance level to *P *= 0.025 (= 0.05/2). In addition, the follicular diameters of day 0 were also compared between groups (FSH- vs. +, and IC- vs. +) to check if there was no preexisting difference between them. The software JMP version 9 (SAS Institute, Cary, North Carolina) was used for these statistical analyses.

## Results

### Three-dimensional follicle culture system

#### Effects of collagen gel culture on follicular growth

**Figure 1:**
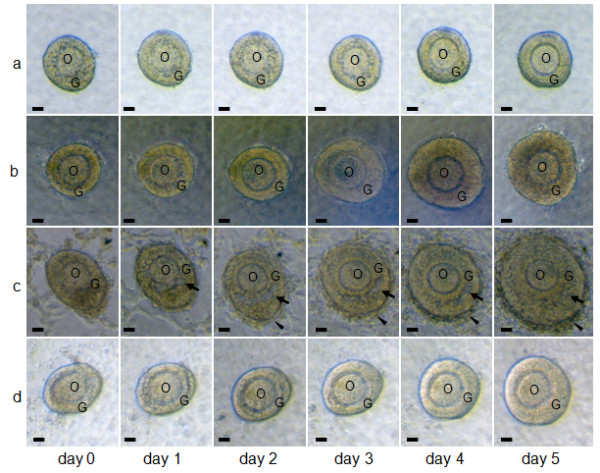
**Effects of FSH, interstitial cells, and granulosa cells on follicular growth in the three-dimensional follicle culture**. Follicles cultured in collagen gel (a) without both FSH and interstitial cells, (b) with FSH and without interstitial cells, (c) with both FSH and interstitial cells, and (d) with both FSH and granulosa cells for 5 days. Arrows in (c) show the follicular antrum-like cavity. The arrowheads in (c) show a cell layer formed in the outermost part of the follicle during culture. O, oocyte; G, granulosa cells. Scale bars = 25 μm.

The follicles used in this culture system were obtained from 10- to 14-day-old mice and were approximately 140 μm in diameter. Their stage of development was approximately secondary to preantral follicles, and they were surrounded by a layer of theca cells (Figure 1a).

When cultured in the liquid medium, the follicles did not develop, but they shrank (data not shown). The theca cells in the outermost part of the follicles escaped and adhered to the bottom of the culture dish. In addition, granulosa cells broke away from the oocyte and spread through the basement membrane. Therefore, the follicle structure in the liquid medium became completely different from that in ovaries. We used collagen gel in order to resolve this problem and maintain the three-dimensional structure of the follicle during culture. As shown in Figure 1a, the follicles maintained their structure in these gels, with their appearance being similar to that in ovaries. The granulosa cells stayed around the oocyte, and the basement membrane surrounded the follicle. Moreover, the follicles grew slightly during culture although the theca cell layer in the outermost part of the follicle did not develop during this time.

#### Effect of FSH on follicular growth

**Figure 2:**
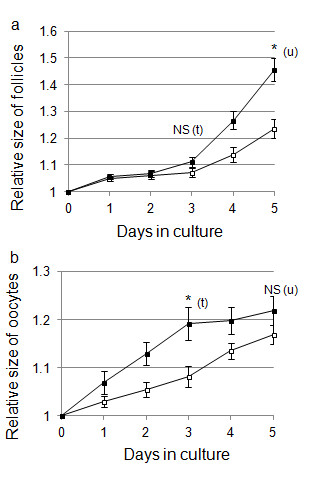
**Effect of FSH on the growth of follicles and oocytes during culture**. (a) Changes in the size of follicles during culture in collagen gel. Follicle size is expressed as follicle diameter relative to the size on day 0 (mean ± SE). The actual size of follicles cultured with FSH is 129.5 ± 2.5 μm and without FSH is 123.9 ± 2.6 μm. Black square show follicles cultured with FSH (n = 21) and white square show follicles cultured without FSH (n = 26). (b) Changes in the size of oocytes during culture. Oocyte size is expressed as oocyte diameter relative to the size on day 0 (mean ± SE). Black square show oocytes cultured with FSH (n = 15) and white square show oocytes cultured without FSH (n = 14). (u), Mann-Whitney U test; (t), t-test; * significance (*P *< 0.025); NS, not significant.

Because it has been suggested that FSH may play a role in follicular growth, we examined the effects of FSH on follicular development in collagen gels for 5 days. Follicles cultured in collagen gel in the presence of FSH (Figure 1b) grew and maintained their three-dimensional shape during culture and were larger than those grown in the absence of FSH (Figure 1a). Figure 2 shows the effects of FSH on the growth of the follicles (Figure 2a) and oocyte (Figure 2b). The actual size of follicles were 129.5 ± 2.5 μm (presence of FSH) and 123.9 ± 2.6 μm (absence of FSH), and the difference was not significant between these groups on day 0 (*t *= 1.60, *P *> 0.025). On day 3, the relative sizes of the follicles with FSH and without FSH were not significantly different (Figure 2a; *t *= 1.48, *P *> 0.025). On day 5, the diameters of the follicles grown in the presence or absence of FSH were 145% and 125% of the values at day 0, respectively. On day 5, the relative size of the follicles grown in the presence of FSH was significantly larger than that of the follicles grown in the absence of FSH (Figure 2a; *U *= 454.06, *P *< 0.025). On day 3, the diameters of the oocytes grown in the presence or absence of FSH were 119% and 108% of day 0 values, respectively. FSH induced significant increases in oocyte growth during the first 3 days (Figure 2b; *t *= 2.78, *P *< 0.025) although these stimulating effects became obscure on day 5 (Figure 2b; *U *= 62.99, *P *> 0.025). On the basis of these observations, FSH was always included in the culture medium in all the following experiments.

#### Co-culture of follicles with interstitial cells

Although the follicles maintained their three-dimensional structure in collagen gel, the development of theca cell layers was not observed (Figure 1a, b). As it has been suggested that interstitial cells gather around follicles for theca cell layer formation, we considered that theca cell layer formation may be reproduced by co-culturing follicles and interstitial cells.

**Figure 3:**
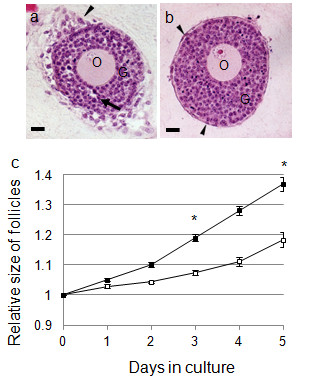
**Effects of the co-culture of follicles and interstitial cells on follicular growth**. (a, b) HE-stained images of follicles. Follicles were cultured for 5 days with (a) and without (b) interstitial cells, paraffin-sectioned, and HE-stained. The arrows in (a) show a follicular antrum-like cavity, while the arrowheads in (a) and (b) show cell layers in the outermost part of follicles during culture. O, oocyte; G, granulosa cells. Scale bars = 25 μm. (c) Changes in the size of follicles during culture. Follicle growth is expressed as follicle diameter relative to the size on day 0 (mean ± SE). The actual size of follicles co-cultured with interstitial cells is 123.6 ± 1.42 μm and without interstitial cells is 118.0 ± 2.33 μm. Black square show follicles co-cultured with interstitial cells (n = 84) and white square show follicles cultured without interstitial cells (n = 28). * significance (*P *< 0.025, Mann-Whitney U test).

When the follicles were co-cultured with interstitial cells, the development of cell layers in the outermost part of the follicle could be observed on day 2. This layer developed further during the culture period (Figure 1c; arrowheads). In addition, follicular antrum-like cavities were observed in the granulosa cell layers of some follicles co-cultured with interstitial cells (Figure 1c; arrows). These characteristics (i.e., development of theca-cell layer-like multiple cell layers and follicular antrum-like cavities) were clearly observed in the paraffin section of follicles co-cultured with interstitial cells (Figure 3a). However, in follicles cultured without interstitial cells, the cell layer in the outermost part of the follicle remained as a single layer (Figure 3b; arrows), while the cavities in the granulosa cell layers were not observed despite an increase in the number of these cells (Figure 3b). The actual size of follicles were 123.6 ± 1.42 μm (cultured with IC) and 118.0 ± 2.33 μm (cultured without IC), and the difference was not significant between these groups on day 0 (*t *= -1.96, *P *> 0.025). From day 3, the diameter of the follicles was significantly greater in the co-cultured group than in the group cultured alone (Figure 3c; *U *= -4.87, *P *< 0.025).

#### Co-culture of follicles with granulosa cells

In order to examine the possible involvement of granulosa cells in theca cell layer formation, we co-cultured the follicles with granulosa cells. The follicles co-cultured with granulosa cells grew and maintained their structure, but the development of a cell layer in the outermost part of the follicle or formation of cavities in granulosa cell layers were not observed (Figure 1d).

### Composition of isolated interstitial cell suspension

**Figure 4:**
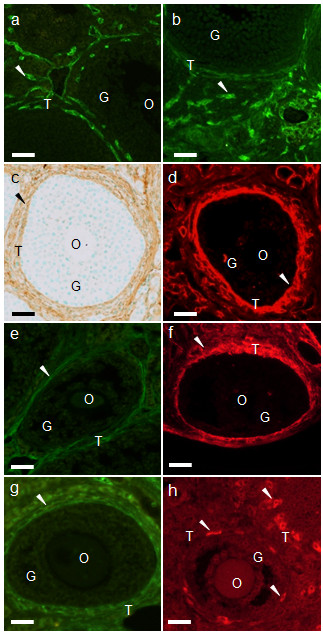
**Immunohistochemical localization of the cell markers in mouse ovaries**. Immunohistochemistry of (a) Flk-1, (b) F4/80, (c) fibronectin, (d) laminin, (e) tenascin, (f) collagen type IV, (g) THY1, and (h) CYP 17A-1 in mouse ovaries. (a, b, d-h) visualized by immunofluorescence and (c) visualized by ABC staining. O, oocyte; G, granulosa cells; T, theca cells. Scale bars = 25 μm.

Ovaries contain not only interstitial cells but also oocytes, granulosa cells, endothelial cells, macrophages, and theca cells. We therefore examined the purity of the interstitial cells used in the co-culture experiment by examining the localization of various cell markers. Figure 4 shows the distribution of the cell markers observed in the ovaries. Flk-1 and F4/80, established markers of endothelial cells (Figure 4a) and macrophages (Figure 4b), respectively, were observed in the stroma of the ovaries. Fibronectin was localized in the theca and interstitial cells (Figure 4c), while laminin was localized in the basement membrane and theca, interstitial, and endothelial cells (Figure 4d). These results indicated that the fibronectin-positive cells or laminin-positive/Flk-1 negative cells in mouse ovaries could be classified as theca-interstitial cells (Table 2). Because of the difficulty in distinguishing theca and interstitial cells, each of which are believed to differentiate into the other cell type, these two cell types were classified as theca-interstitial cells in this study.

**Table 2:** Comparison of cell marker localization in isolated cells from ovaries

In ovaries						Cell suspension	
marker	**Oo**	**IC**	**TC**	**EC**	**Ma**	**GC**	**IC**
fibronectin	-	+	+	-	-	-	61.0% (891/1461)
laminin	-	+	+	+	-	-	88.1% (378/429)
Flk-1	-	-	-	+	-	-	0% (0/1500)
F4/80	-	-	-	-	+	-	0.3% (3/895)

The staining of markers in the cells isolated from ovaries is shown as + (positive) or − (negative). The proportion of cells showing a positive signal in interstitial cell suspensions is expressed as a percentage. The numbers in parenthesis are the number of positive cells per total counted cells. Oo: oocyte, IC: interstitial cells, TC: theca cells, EC: endothelial cells, Ma: macrophages, and GC: granulosa cells.

The proportion of the cells with these cell markers in the interstitial cell suspension prepared for co-culture experiments is shown in Table 2. Fibronectin was observed in 61.0% of the cells, while laminin was observed in 88.1% of the cells in the interstitial cell suspension. The markers for endothelial cell (Flk-1) and macrophage (F4/80) were almost completely absent from the interstitial cell suspension. Therefore, the percentage of theca-interstitial cells in the prepared cell suspension should be approximately between 61.0% (proportion of fibronectin-positive cells) and 88.1% (proportion of laminin-positive and Flk-1 negative cells).

As the GC suspension was negative for all the cell markers in our experiments (Figure 4, Table 2), it is possible that the remaining cell marker-negative cells (12-39%) represent the granulosa cells contaminating the interstitial cell suspension. We confirmed that the purified GC suspension was negative for all cell markers (data not shown).

### Three-dimensional shape of the cultured follicles

**Figure 5:**
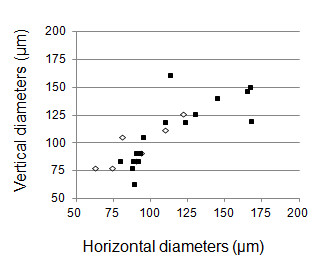
**Relationship between the horizontal and vertical diameters of cultured follicles**. The horizontal line shows horizontal follicle diameters at day 5, and the vertical line shows vertical follicle diameters on day 5. Black square show follicles co-cultured with interstitial cells (n = 21) and white square show follicles cultured without interstitial cells (n = 7).

To investigate whether the follicles cultured in collagen gel maintained their three-dimensional shape, the follicles were fixed after 5 days of culture and sectioned serially in the horizontal plane. The largest section was chosen from all serial sections, and its diameter was designated as the "horizontal diameter." The numbers of serial sections were counted, and the "vertical diameter" was calculated by multiplying the thickness of the sections by the number of sections. The vertical and horizontal diameters of the follicles were nearly equal irrespective of whether the follicles were co-cultured with interstitial cells (Figure 5). The ratio of vertical to horizontal diameters was nearly 1 in both types of culture (presence, 0.97 and absence, 1.07).

### Characterization of the cell layer formed in the outermost part of follicles during culture

In order to determine whether the cells forming layers at the outermost part of the cultured follicles were theca cells, the localization of the markers for these cells was examined in cultured follicles.

We first confirmed the localization of several potential theca cell markers in mouse ovaries (Figure 4c-h). Consistent with previous reports [19-25], the extracellular matrix proteins fibronectin (Figure 4c), laminin (Figure 4d), tenascin (Figure 4e), and collagen type IV (Figure 4f) were detected in the theca cell layers of the follicles in mouse ovaries. As reported previously [18], in addition to these extracellular matrix proteins, theca cell layers included the cell adhesion molecule THY1 (Figure 4g). In addition, the steroid synthesizing enzyme CYP17A-1, which is reported to be localized only in the theca cells in ovaries [3], was selectively detected in a subpopulation of the theca cells in mouse ovaries (Figure 4h).

**Figure 6:**
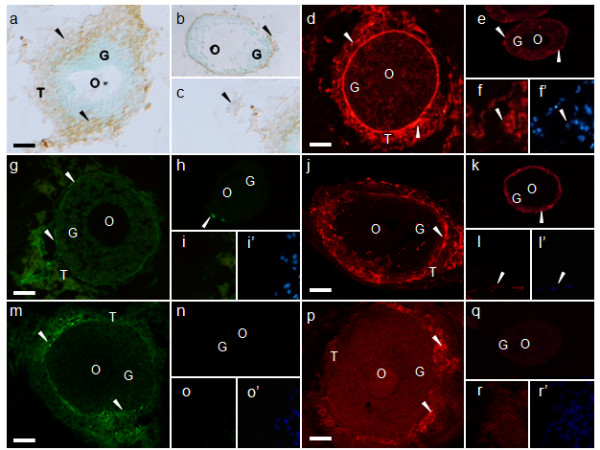
**Immunohistochemical localization of the theca cell markers in cultured follicles**. Immunohistochemistry of (a-c) fibronectin, (d-f) laminin, (g-i) tenascin, (j-l) collagen type IV, (m-o) THY1, and (p-r) CYP17A-1. (f, f'), (i, i'), (l, l'), (o, o'), and (r, r'), are same view respectively. (f', i', l', o', r') are DAPI staining. (d-r') visualized by immunofluorescence and (a-c) visualized by ABC staining. (a, d, g, j, m, p) were cultured follicles for 5 days, (b, e, h, k, n, q) were cultured follicles for 0 day (isolated follicle), and (c, f, f', i, i', l, l', o, o', r, r') were interstitial cells distanced from follicles. The arrowheads indicate each signal. O, oocyte; G, granulosa cells; T, theca cells. Scale bars = 25 μm.

We then examined the localization of these cell markers within the isolated follicles; there were a few theca cells in isolated follicles. Fibronectin (Figure 6b), laminin (Figure 6e), and collagen type IV (Figure 6k) were localized in basement membrane and theca cells. Tenascin was sometimes observed in theca cells (Figure 6h). However, Thy-1 (Figure 6n) and CYP17A-1 (Figure 6q) were not observed in follicles. The cell layers formed in the outermost part of the cultured follicles were selectively positive for each of the following markers: fibronectin (Figure 6a), laminin (Figure 6d), tenascin (Figure 6g), collagen type IV (Figure 6j), THY1 (Figure 6m), and CYP17A-1 (Figure 6p). In addition, interstitial cells that distanced from follicles were observed. Fibronectin (Figure 6c), laminin (Figure 6f, f'), and collagen IV (Figure 6l, l') were observed, but tenascin (Figure 6 i, i'), Thy-1 (Figure 6 o, o'), and CYP17A-1 (Figure 6 r, r') were not localized in interstitial cells. Taken together, these results confirmed that the cell layers in the outermost part of the follicles in co-culture may differentiate into theca cells.

## Discussion

Follicles are constructed from oocytes, granulosa cells, and theca cells, with interactions between these cells, and other constituents being important for folliculogenesis. Studies on folliculogenesis and follicular functions have been conducted using several follicle culture systems. However, these culture systems do not reproduce theca cell layer formation. The mechanism of theca cell layer formation has therefore not been clarified adequately. To examine how the theca cell layer formed, a follicle culture system that maintains the three-dimensional shape of follicles and reproduces theca cell layer formation was needed. In this study, we established a follicle culture system that satisfied these requirements.

### Establishment of follicle culture system that can reproduce theca cell layer formation

#### Maintenance of follicle structure during culture

In our follicle culture system, collagen gel was used in order to maintain follicle structure. We confirmed that the follicles maintained their three-dimensional shapes when cultured in collagen gel by measuring the vertical and horizontal diameters of serial sections (Figure 5), and using confocal microscopy [18]. Some follicle culture systems utilize collagen gel as the matrix. It was reported that bovine late preantral follicles, attached to a fragment of ovary containing interstitial cells and cultured in collagen gel, maintained follicle structure and formed a follicular antrum [17]. Furthermore, it has been shown that mouse follicles, approximately 140 μm in diameter, cultured in collagen gel maintained their three-dimensional shape during growth [17]. Both these follicle culture systems used type I collagen as the gel matrix. Type I collagen is one of the extracellular matrices that is present in adequate quantities throughout ovaries [20,23,24,26-28]. It has been suggested that culturing follicles in type I collagen may provide the follicles with an environment similar to that in the ovary; moreover, type I collagen plays an important role in the growth of secondary and preantral follicles during culture [16].

#### Effects of FSH on follicular growth

FSH is indispensable for the proliferation of granulosa cells, maturation of oocytes, and production of estradiol in culture systems [8,10-12,18,29,30]. In the ovaries of FSH-beta knockout mice, follicular growth was arrested at the preantral stage; therefore, neither antral formation nor ovulation occurred [31].

In our follicle culture system, the relative size of follicles in the presence of FSH was significantly greater than that in its absence (Figure 2a), indicating that FSH was effective in folliculogenesis on day 5. The effect of FSH on follicular development was also apparent during the later phase of the culture period (i.e., from day 3 onward). The FSH receptor is expressed in granulosa cells, and follicles are sensitive to FSH after the preantral stage [32]. The follicles used in our culture system were secondary to the early preantral stage. FSH may therefore cause granulosa cells to proliferate after the preantral stage in this follicle culture system.

The effect of FSH on oocytes was different from that on follicles. In our follicle culture system, the diameter of oocytes on day 3 was significantly greater in the presence of FSH than in its absence, although this difference became insignificant on day 5 (Figure 2b). Similarly, Thomas et al. [33] reported that in a mouse oocyte-granulosa cell complex culture system, FSH increased oocyte diameter on day 3, but this effect of the hormone became insignificant on day 7. There is evidence that oocytes grow most quickly during early follicular development and reach their maximum sizes until the preantral follicular stage [32]. As oocytes can reach their maximum size in the absence of FSH, it appears that FSH may accelerate the speed of oocyte growth *in vitro*. FSH receptors are expressed in oocytes although the direct effect of FSH on these cells remains unclear [34]. It has been shown that oocytes and granulosa cells are connected by a gap junction, and some paracrine factors are secreted between the cells to regulate oocyte growth [35]. FSH may act on granulosa cells and consequently reinforce the relationship between these cells and oocytes. However, whether FSH acts directly on oocytes or through granulosa cells has not been established.

LH is also important for follicular development, especially the steroid production, ovulation, and corpus luteum formation. However, in LH β null mice ovary, theca cell layers develop and the theca cell markers (*Lhcgr*, *Bmp4*, and *Cyp17a1*) are expressed [36]. In this study, we aim to establish the follicle culture system for examine the process of theca cell layer formation. Therefore, the effects of LH on follicular development were not examined in this study. It is considered that LH addition must be tried for the progress of this follicle culture system.

#### Effects of the co-culturing of follicles and interstitial cells on follicular growth

When follicles were cultured alone, theca cell layers did not develop (Figure 1b). In contrast, the follicles co-cultured with interstitial cells formed cell layers at their outermost part (Figure 1c); this is confirmed as the theca cell layer based on the localization of various cell markers (Figure 6). Moreover, follicles cultured with interstitial cells grew larger than those cultured alone (Figure 3c). These observations suggest that interstitial cells are important for theca cell layer formation and follicle growth.

In some follicles cultured with interstitial cells, follicular antrum-like cavities were formed in the granulosa cell layer, but no such cavities were formed in follicles cultured alone (Figure 1b, 3b). In ovaries, the water required to form the follicular fluid present in the follicular antrum is supplied to the granulosa cell layers by the capillaries present in theca cell layers through the basement membrane [37]. Moreover, the composition of the extracellular matrix in basement membranes and the theca cell layer changes during follicular development [20,25-28,38]; these changes affect the water permeability of the basement membrane and hence water flow into granulosa cell layers [37]. We therefore suspect that theca cell layer formation may be involved in the formation of follicular antrum-like cavities. Theca cell layer formation and formation of antrum-like cavities were observed in co-cultured follicles only. This implies that the theca cell layer may be necessary to change the composition of the basement membrane extracellular matrix and increase its permeability to water.

The formation and development of the follicular antrum-like cavity in follicles co-cultured with interstitial cells may contribute to their growth rates. However, the cavities formed in follicles co-cultured with interstitial cells were smaller than the follicular antrum observed in the ovarian follicles. The absence of capillaries in our follicle culture system may be responsible for this difference. In addition, if follicles cultured more than 5 days, this antrum-like cavity might increase the volume. This is very interesting problem, and by using this follicle culture system, the process of antrum formation might be elucidated.

### Mechanisms of theca cell layer formation in the follicle culture system

#### Aggregation of interstitial cells

Mammalian follicles are composed of an oocyte, granulosa cells, and theca cells. Of these cells, theca cells are not observed in primordial follicles but appear in secondary follicles. It is thought that theca cells are formed from interstitial cells aggregating around follicles although this process has not been examined experimentally [1,2]. Our follicle culture system raised the possibility that interstitial cell aggregation may contribute to theca cell layer formation (Figure 1c). The interstitial cell suspension used for co-culturing experiments contained 21.9-39.0% of contaminating granulosa cells (Table 2). However, theca cell layer formation was not observed in the follicles co-cultured with granulosa cells (Figure 1d), suggesting that follicles may preferentially attract interstitial cells, and cell layer formation was accomplished by interstitial cells.

#### Differentiation of interstitial cells to theca cells

Our study confirmed that theca cell markers localized in the cell layers formed in the outermost part of the follicle during culture (Figure 6). However, the cell marker except for extracellular matrix were not observed in the isolated follicles (did not culture) and interstitial cells (Figure 6). Tajima et al. [6] and Orisaka et al. [7] reported that interstitial cells co-cultured with granulosa cells (the two types of cells were on opposite sides of the collagen membrane) became spindle-shapes and constructed multiple layers similar to the theca cell layers in the ovary. In addition, androgen production in the theca cells increased. It is therefore plausible that interstitial cells differentiate into theca cells, and this differentiation may be promoted by interaction with granulosa cells [6,7]. In addition, it has been suggested that interaction of the Kit ligand, produced by granulosa cells, and c-kit, present in interstitial cells, may induce differentiation of interstitial cells into theca cells [39]. In the ovaries of mice with a mutated Kit ligand gene, folliculogenesis was arrested at the beginning of theca cell formation [40]. These observations suggest that interactions of theca and granulosa cells may be important for theca cell differentiation. However, the factors involved in theca cell differentiation have not been identified. Our follicle culture system may be suitable for elucidating the roles of granulosa cells in theca cell layer formation.

## Conclusions

In this study, we established a three-dimensional follicle culture system. Using this culture system, follicles could (i) maintain their three-dimensional shape by being embedded in collagen gel, (ii) increase their size following stimulation by FSH, and (iii) reproduce theca cell layer formation when co-cultured with interstitial cells. This follicle culture system therefore has the potential to elucidate the mechanisms of theca cell layer formation and investigate many unresolved phenomena concerning folliculogenesis.

## Competing interests

The authors declare that they have no competing interests.

## Authors' contributions

SI participated in the design of the study, carried out all experiments, and drafted the manuscript. YY, CM, and SH helped development of follicle culture system. AS and ST helped to draft the manuscript. KY conceived and coordinated this study, and helped to draft the manuscript. All authors read and approved the final manuscript.
